# Regulatory T cell dysfunction and immunotherapeutic breakthroughs in type 1 diabetes

**DOI:** 10.3389/fendo.2025.1740102

**Published:** 2026-01-06

**Authors:** Kuang-Ji Zhou, Shan-Jie Rong, Yue-Chen Liu, Fei Sun, Ting Wang, Qi-Lin Yu, Cong-Yi Wang

**Affiliations:** 1 Department of Respiratory and Critical Care Medicine, The Center for Biomedical Research, National Health Commission (NHC) Key Laboratory of Respiratory Diseases, Tongji Hospital, Tongji Medical College, Huazhong University of Science and Technology, Wuhan, China; 2 Tongji Shanxi Hospital, Shanxi Bethune Hospital, Shanxi Academy of Medical Science, Third Hospital of Shanxi Medical University, The Key Laboratory of Endocrine and Metabolic Diseases of Shanxi Province, Taiyuan, China; 3 Diabetes Research Center, Qatar Biomedical Research Institute, Hamad Bin Khalifa University, Doha, Qatar

**Keywords:** pancreas resident Treg, regulatory T cells (Tregs), Treg-based immunotherapy, Treg dysfunction, type 1 diabetes (T1D)

## Abstract

Type 1 diabetes (T1D) is characterized by the autoimmune destruction of insulin-producing β-cells in the pancreas. Regulatory T cells (Tregs) are essential for maintaining immune tolerance, but they manifest impaired functionality, particularly within the pancreatic microenvironment, during T1D development. This review aimed to discuss Treg biology including the developmental trajectory, phenotypic heterogeneity, and suppressive function, by which we sought to emphasize their compromised role in T1D pathogenesis associated with genetic/epigenetic factors along with impaired cytokine signaling. The unique chemokine receptor expression signature, migratory capacity, and metabolic adaptation of pancreatic Tregs are highlighted, alongside insights from single-cell studies. The evolution of Treg-based immunotherapies is explored, with emphasis on genetically engineered Tregs (EngTregs), which are designed for the stable ectopic expression of FoxP3 and antigen-specific receptors, such as T cell receptors (TCR) or chimeric antigen receptors (CAR). It also highlights advancements in genome-editing and delivery technologies, along with rationally designed combination strategies incorporated into multifunctional cellular products. Despite encouraging preclinical results, significant challenges persist in clinical translation. Overall, this review synthesizes existing knowledge and outlines future directions in Treg biology and immunotherapy, underscoring the potential of next-generation Treg therapy to achieve durable immune tolerance in T1D.

## 1 Introduction

Type 1 diabetes (T1D) is caused by autoimmune-mediated destruction of the pancreatic β-cells, ultimately leading to an absolute deficiency of insulin. The global incidence of T1D is increasing at an annual rate of 3-5%, which is more evident in children (1). T1D pathogenesis is driven by the interplay between genetic susceptibility (e.g., HLA class II genes) and environmental factors (e.g., viral infections, gut dysbiosis) (2). Recent studies underscore the role of the gut microbiome (such as enteroviruses) in triggering the generation of islet autoantibodies, suggesting that early environmental exposures may influence immune responses in later life (2, 3). A core immunological hallmark of T1D is the imbalance between effector T cells (Teffs) and regulatory T cells (Tregs). Teffs mediate β-cell damage through the recognition of islet autoantigens (e.g., insulin, GAD65), while functional impairment or reduced numbers of Tregs further exacerbate the breakdown of self-tolerance (4, 5). In general, Tregs rely on the transcription factor FoxP3 to sustain their identity, which warrants their capability to suppress Teff activity by producing CTLA-4, IL-10, TGF-β and so on. Tregs exhibit significant heterogeneity, with subpopulations diversely generated in distinct anatomical sites and functional states (6). The stability of Tregs is influenced by epigenetic factors (e.g., the DNA methylation status of FoxP3 locus) and metabolic pathways (e.g., mTOR signaling), while IL-2 signaling is critical for their survival and function (7, 8).

**Figure 1:**
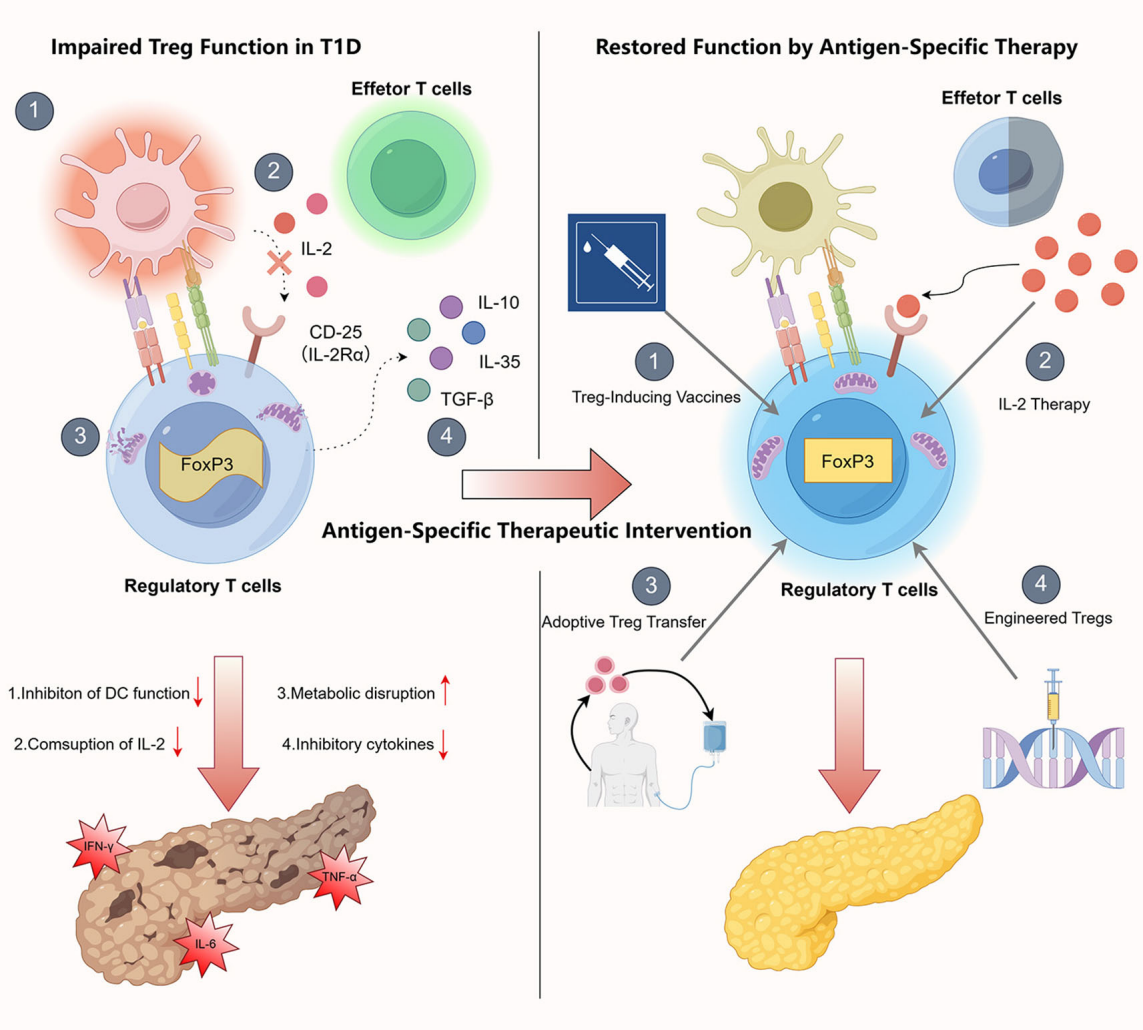
Dysfunctional Tregs in T1D and their restoration via antigen-specific therapies. In T1D, impaired Treg function is a key factor in the breakdown of immune tolerance. This dysfunction involves several mechanisms: First, Tregs fail to effectively remove co-stimulatory signals from the surface of APCs, leading to sustained APC activation and enhanced stimulation of autoreactive Teff. Second, reduced expression or function of CD25 (IL-2Rα) on Tregs compromises their ability to compete for IL-2 produced by conventional T cells, thereby impairing Treg survival and functional maintenance. Third, mitochondrial metabolic abnormalities in Tregs—such as diminished oxidative phosphorylation—undermine their suppressive activity and adaptability in inflammatory environments. Finally, Tregs exhibit reduced secretion of inhibitory cytokines, including IL-10, TGF-β, and IL-35, which weakens their control over Teff activation and inflammatory responses, thereby accelerating autoimmune damage to insulin-producing β cells. To restore immune homeostasis, antigen-specific Treg-targeted therapies have been developed in recent years. These include Treg-inducing vaccines that expand antigen-specific Tregs under tolerogenic conditions; low-dose IL-2 therapy to enhance Treg signaling and function; adoptive Treg transfer, aimed at improving *in vivo* persistence and tissue homing; and genetically engineered Tregs—such as those expressing chimeric antigen receptors (CARs) or specific T cell receptors (TCRs) targeting pancreatic antigens—to increase treatment precision and efficacy. Each of these strategies presents distinct advantages and limitations in terms of specificity, durability, safety, and scalability, which will be systematically discussed in the main text.

The pancreatic microenvironment uniquely regulates the function and homing of Tregs. Tissue-resident Tregs (trTregs) characterized by highly expressed chemokine receptors (e.g., CCR5), localize around pancreatic islets, where they interact with local antigen-presenting cells (APCs) to suppress autoimmune responses (9). However, in T1D patients, pancreatic Tregs are generally featured by the destabilized FoxP3 expression along with ectopic expression of inflammatory cytokines (e.g., IL-6, IFN-γ) to compromise their suppressive capacity (10). Recent single-cell sequencing studies have unveiled heterogeneity within the pancreatic Treg populations, identifying subsets with distinct β-cell protective metabolic signatures (11). We, therefore, in this review, seek to comprehensively discuss the regulation of Treg development, stability, classification, and functional mechanisms. We will also specifically explore the mechanisms underlying Treg dysfunction in T1D settings, critically assess the adaptive features of tissue-specific Tregs, and systematically review the latest advances and future directions in Treg-based immunotherapies (**Figure 1**).

## 2 Treg biology

### 2.1 Lineage heterogeneity: developmental origins and functional subsets

Tregs serve as a central regulator of immune homeostasis, with their complex heterogeneity stemming from diverse developmental origins, phenotypic characteristics, and functional mechanisms. Characterized by high immunosuppressive capacity, Tregs constitute approximately 10% of CD4^+^ T cells in healthy individuals. They are defined by the expression of transcription factor FoxP3 (8), and are critical for controlling immune responses and maintaining peripheral tolerance (12–15). In humans, Tregs are often defined by the combined expression of CD3^+^CD4^+^CD25^hi^FOXP3^+^CD127^low/−^ (16). Based on the expression patterns of FoxP3, CD25, and CD45RA, they can be further divided into three distinct populations. Fraction I (Fr. I) comprises CD45RA^+^FoxP3^lo^CD25^lo^ naive Tregs, which are considered bona fide Tregs characterized by a demethylated Treg-specific demethylated region (TSDR) and full suppressive capacity. Upon activation, nTregs differentiate into Fraction II (Fr. II), the effector Tregs, which exhibit a CD45RA^-^FOXP3^hi^ CD25^hi^ phenotype and possess potent suppressive activity. In contrast, Fraction III (Fr. III; CD45RA^-^FOXP3^lo^CD25^lo^) represents a highly heterogeneous population composed of a mixture of genuine Treg and non-Treg cells (17). Recent studies have successfully refined this subset using additional surface markers such as CD127, CCR4, and CD49d. For instance, the CD127^+^CD25^lo^ subpopulation exhibits a fully methylated TSDR and high inflammatory cytokine production, which categorized them as non-Tregs. Furthermore, the CD49d^+^CCR4^−^ phenotype can distinguish an inflammatory subpopulation capable of secreting IFN-γ and IL-17. Importantly, circulating T follicular regulatory (cTfr) cells are also found within Fr. III. These cells show stable FoxP3 expression, a demethylated TSDR, and suppressive function, confirming their identity as authentic Tregs and accounting for about 30% of Fr. III cells in healthy individuals (18). Therefore, Fr. III should no longer be considered as a uniform entity but must be precisely delineated using these newly defined markers (19). However, human and mouse Tregs are heterogeneous in terms of developmental origin, functional activity, and activation status. Based on cellular origin, Tregs are classified into two main types: thymus-derived Tregs (tTregs), which develop in the thymus and comprise the majority of the Treg pool in secondary lymphoid organs. By contrast, peripherally induced Tregs (pTregs) originate from naive FoxP3^−^CD4^+^ T cells at inflammatory sites in the periphery where they acquire FoxP3 expression (20). Conventional T cells (Tconv) can also differentiate *in vitro* to form induced Tregs (iTregs), though these cells typically lack the complete epigenetic programming for stable Treg gene expression and exhibit functional instability (20, 21). Therefore, the pTreg category encompasses both *in vivo*-derived cells and *in vitro*-generated iTregs (20).

Tregs can be further categorized by activation state into natural/thymic Tregs (nTregs), activated Tregs (aTregs), and effector Tregs (eTregs). CD62L^+^CD44^−^TCF1^+^ nTregs stimulated by TCR signaling in the presence of IL-2 transit into CD62L^−^CD44^(mid/hi)^TCF1^+^ aTregs, which subsequently differentiate into CD62L^−^CD44^hi^TCF1^−^ eTregs (20). Although the newly produced nTregs possess suboptimal immunosuppressive capacity, upon TCR engagement in lymph nodes, they proliferate and differentiate into highly suppressive eTregs. These eTregs suppress the antigen-specific maturation of APCs such as dendritic cells (DCs) (22). Conversely, eTregs deplete IL-2 through increased sensitivity to the IL-2 receptor and secrete inhibitory cytokines including IL-10, TGF-β, and IL-35, thereby suppressing immune function in an antigen-nonspecific manner (23). A specialized Treg subset, type 1 regulatory T (Tr1) cells (FoxP3^−^IL-10^+^ CD4^+^ T cells), do not express FoxP3 but secrete high levels of IL-10, playing a vital role in promoting peripheral tolerance (24). Notably, elevated levels of Tr1 cells are associated with better management of blood glucose levels in new-onset T1D patients (25), and preclinical studies in autoimmune mice confirm their protection can be harnessed through antigen-specific induction via peptide-MHC-coated nanoparticles (26). These findings suggest that a strategy of fostering antigen-specific Treg populations directly within the patient could be a viable way to exploit Tr1 cells for therapy (27).

### 2.2 Molecular determinants: identity and stability markers

Key molecular markers of Tregs not only establish their cellular identity but are also critical determinants of their functional stability and suppressive capacity. The most canonical identity markers include FoxP3, the critical transcription factor, and surface markers such as CD25 and CTLA-4 (8). Among which, FoxP3 is indispensable for Treg function (12, 28, 29). Loss-of-function mutations in FoxP3 cause fatal autoimmune pathology. In scurfy mice, such mutations lead to severe systemic autoimmunity, while in humans, FoxP3 mutations result in functional Treg deficiency and cause Immune dysregulation, Polyendocrinopathy, Enteropathy, and X-linked (IPEX) syndrome. IPEX manifests as multi-organ autoimmunity, including diabetes, thyroiditis, and allergies (e.g., eczema), and is typically fatal within the first year of life without bone marrow transplantation (30–32). Prior to FoxP3 expression in early tTreg precursors, the genomic organizer SATB1 (Special AT-rich sequence-binding protein 1) cooperates with unknown pioneer factors to bind super-enhancers governing the FoxP3 locus and other Treg signature genes. This binding establishes a permissive epigenomic landscape essential for the induction of key Treg genes (14).The DNA demethylation occurring during this reprogramming is indispensable for the long-term stability of the Treg phenotype and its suppressive function (33, 34). However, FoxP3 function and the Treg-specific epigenome operate through distinct yet complementary mechanisms (**Table 1**). Of note, the preservation of the TSDR within the Foxp3 CNS2 enhancer is essential for maintaining the stable lineage identity and suppressive function of Tregs. Deletion of CNS2 triggers progressive erosion of FoxP3 expression stability during Treg proliferation, ultimately resulting in cell extinction (35–38). Treg-specific epigenetic alterations not only facilitate the lineage establishment and functional persistence of Tregs, but also serve as a genomic signature to distinguish them from Tconv cells (34, 39). Although both Tregs and activated Tconv cells express CD25 and CTLA-4, but Tregs constitutively express these molecules at high levels, while they only express in Tconv cells upon TCR stimulation. This disparity is mechanistically linked to unique CpG methylation profiles in the TSDR of a panel of genes, including FoxP3, CD25, CTLA-4, and Helios (34). Hence, a hypomethylated epigenome is a vital criterion for delineating functionally competent Tregs, safeguarding their identity amid fluctuations in FoxP3 expression.

**Table 1:** Dual mechanisms of FoxP3 and epigenetic regulation in shaping treg identity: independence and synergy.

Relationship category	Core finding/Mechanism	Specific fvidence/Examples	Functional significance/Impact		Nature of relationship
Independence	FoxP3 expression is insufficient to induce Treg-specific epigenetic changes.	FoxP3 overexpression fails to induce Treg-specific DNA demethylation patterns in Tconv (34).	The formation and maintenance of Treg epigenetic signatures are independent of FoxP3.		FoxP3-independent
	FoxP3 deficiency does not abolish core epigenetic signatures in Treg cells.	Treg cells from FoxP3-knockout mice retain DNA demethylation patterns similar to wild-type Tregs (especially in FoxP3 CNS regions) (34).			
	Treg-specific epigenetic changes precede FoxP3 expression, driving lineage commitment (239).	DNA demethylation initiates during early Treg development before FoxP3 expression. Treg-specific super-enhancers (SEs) activate preceding FoxP3 expression. SEs contain Treg-DRs linked to key genes (FoxP3, Il2ra, Ctla4). SE formation is driven by FoxP3-independent factors (e.g., Satb1).	Epigenetic remodeling is a pioneer event that directs lineage specification and lays the regulatory groundwork for FoxP3 expression and function.		Epigenetic priming
Synergy	Treg epigenetic changes act as enhancers to promote/maintain FoxP3 expression.	Key CNS regions in FoxP3 introns function as enhancers, and their demethylation is essential for stable expression (20). DNA demethylation and histone modifications maintain and stabilize the expression of key Treg identity genes, notably FoxP3 (14).	These regulatory elements serve to ensure persistent FoxP3 expression—a cornerstone of Treg lineage stability.		Epigenetic → FoxP3
	FoxP3 recruits epigenetic modifiers to repress non-Treg genes.	FoxP3 recruits HAT/HDAC complexes to suppress T cell activation genes (240). The EOS (Ikaros family) is crucial for FoxP3-mediated gene repression (e.g., IL2), while the histone methyltransferase EZH2 complexes with FoxP3 upon activation to stabilize the Treg transcriptional program during inflammation (241, 242).	Epigenetic mechanisms activate Treg genes (including Foxp3), while FoxP3 represses non-Treg genes, jointly maintaining Treg identity/function.		Bidirectional synergy
	FoxP3 may fine-tune epigenetic features.	FoxP3 binding to specific promoters may indirectly modulate local epigenetic states to enhance Treg stability (15).	FoxP3 potentiates Treg function by dynamically modulating the underlying epigenetic landscape.		FoxP3 → Epigenetic

Other than their lineage-defining transcription factor FoxP3, Treg cells could be further defined by the sustained highly expressed molecules essential for their function, in which, CD25 (IL-2Rα) and CTLA-4 are particularly critical (40–42). CD25 combines with the β and γ chains to form a high-affinity interleukin-2 receptor complex, enabling Treg cells to efficiently sense and consume IL-2 (43, 44), while CTLA-4 serves as a crucial co-inhibitory receptor that empowers them to directly suppress immune activation (45–47). Germline deletion or Treg-specific loss of CTLA-4 leads to fatal autoimmunity resembling that in scurfy mice (47). The core inhibitory mechanisms involve dynamic interactions for signal perception and regulation. A central pathway entails a negative feedback loop related to IL-2 sensing: through the consumption of IL-2 via CD25, Treg cells can rapidly locate and suppress self-reactive T cells early in an immune response, and utilize this cytokine to expand their own population and enhance their suppressive activity, thereby self-reinforcing their regulatory function (48, 49). Strikingly, Treg cells are programmed for antigen-specific suppression during their thymic development. They are not only positively selected by self-antigens but also instructed to mature into functional suppressive cells within the thymus. This pre-established immunological specificity allows them to curb Tconv activation at the onset of an immune response, thereby averting autoimmune pathology (43, 44). Similarly, CTLA-4 mediates “disarming” of APCs, whereby Treg cells physically remove the co-stimulatory molecules CD80/CD86 from the APC surface via trans-endocytosis. This deprivation of co-stimulatory signals not only inhibits immune responses, but also actively dictates the fate of responding T cells. Depending on TCR affinity, high-affinity T cells undergo apoptosis, medium-affinity T cells are induced into an anergic state, and low-affinity T cells remain quiescent. Therefore, Treg cells can orchestrate the survival and outcome of self-reactive T cells, which establishes durable immunosuppressive tolerance. As a result, identifying the mechanisms underlying the reprogramming of Tconv cells into Treg-like suppressors has become a key research objective (50). Even without FoxP3, experimentally eliminating IL-2 production, enforcing constitutive high CTLA-4 expression, and providing TCR stimulation can endow Tconv cells with regulatory functions, indicating that these molecular events constitute pivotal mechanisms of Treg-mediated suppression, which is essential for the prevention of autoimmunity (51).

### 2.3 Suppressive machinery: mechanisms of immune regulation

Tregs regulate immune responses through multiple cell-contact-dependent and -independent mechanisms. In general, Treg cells broadly utilize mechanisms like IL-2 consumption and expression of CTLA4, CD73, or CD39. The induction of alternative pathways including the production of IL-10, TGF-β, IL-35, or indoleamine 2,3-dioxygenase activity, however, is often restricted to particular microenvironments or triggered by specific extracellular cues (47–49). Importantly, many inhibitory mechanisms rely on antigen-specific interactions between Tregs and APCs. High-intensity sustained contact between Tregs and APCs can physically sequester pMHC and CD80/86 co-stimulatory molecules on APC surfaces, thereby disrupting the ability of DCs to interact with and activate cognate effector T cells (45, 46). Additionally, Tregs can secrete apoptotic mediators such as granzymes and perforin to eliminate APCs, conventional T cells, and natural killer (NK) cells (50, 52, 53). Notably, Integral to most Treg functions (54), TCR activation thereby affirms that antigen specificity is indispensable for optimal suppressive efficacy. Studies also indicate that Treg internalization of pMHC requires antigen specificity sharing with the suppressed effector T cells (46). This is particularly critical in T1D, where effector T cells targeting neoepitopes often lack corresponding antigen-specific Tregs for their control (27).

Collectively, Treg heterogeneity arises from diverse developmental origins and functional states, underpinned by distinct molecular identity markers. Defining these identity signatures is critical, not only to resolve functionally discrete Treg subpopulations but also to inform the rational design of antigen-specific Treg therapies. Core molecules like FoxP3 (stabilized by TSDR demethylation), CD25, and CTLA-4 establish Treg identity while enabling potent immunosuppression. Critically, TCR activation is indispensable for Treg-mediated suppression. However, in T1D settings, frequent deficits in cognate antigen recognition specifically impair this process, underscoring the therapeutic imperative for antigen-specific interventions.

## 3 Treg dysfunction in T1D pathogenesis

In the pathogenesis of T1D, alterations in Treg cell quantity are not the central issue, rather their dysfunction, particularly functional impairment within the pancreatic microenvironment, is key to the breakdown of immune tolerance. In pediatric T1D patients, elevated Treg levels are linked to better clinical outcomes, including improved glycemic control (lower HbA1c) and a reduced need for exogenous insulin, suggesting a beneficial role for Tregs in disease management (53). T1D progression was initially thought to be associated with a reduction in Treg cell numbers (54). However, with the advent of more precise methods to distinguish Tregs from Tconv cells and improved Treg quantification techniques, studies revealed no significant change in Treg numbers (55, 56). Advances in epigenetics and transcriptomic analysis have increasingly provided evidence pointing toward Treg dysfunction in T1D. This concept is strongly corroborated by functional evidence from murine models, coupled with analyses of blood samples from individuals with T1D. Research indicates that Tregs from T1D patients exhibit significantly diminished functionality in suppressing autoreactive T cells and inducing immune tolerance. This functional impairment may be linked to reduced Treg stability, particularly the unstable expression of FoxP3 (57, 58). Furthermore, Tregs demonstrate a markedly diminished suppressive capacity within the pancreatic lymph nodes, contributing to exacerbated local inflammation (58).

Undoubtedly, genetic susceptibility has a profound impact on T1D pathogenesis. The majority of susceptible single nucleotide polymorphisms (SNPs) are located near immune genes, indicating a strong association between immune dysregulation and genes crucial for Treg function (59).The most prominent examples include IL2RA, IL2, PTPN2, CTLA4 and IL10 (60). However, a clear connection between these SNPs and functional effects is rarely established. This gap is further complicated by the fact that many implicated genes are critical for both effector and regulatory T cell functions, which poses a challenge for predicting how allelic variants will distinctly influence each population. Nevertheless, the discoveries of SNP-linked functional impairments in Tregs, most notably in IL-2 signaling from human studies (61–63), and corroborated by defective IL-2 responses in Tregs of non-obese diabetic (NOD) mice, have driven initiatives to create therapies focused on this pathway. Unfortunately, low-dose IL-2 therapy, aimed at expanding Tregs, has been associated with adverse effects such as increases in eosinophils and NK cells, as well as reduced C-peptide levels (64).

Epigenetic studies further revealed that autoimmune susceptibility SNPs are enriched in Treg-specific regulatory regions, suggesting their impact on Treg development and function may be greater than on effector T cells. A recent study employed cell type-specific epigenetic profiling in T cells, mapping autoimmune susceptibility SNPs to enhancer regions essential for Treg function (65). Comparative epigenetic profiling between Treg and Tconv populations demonstrated that autoimmune SNPs are enriched within regions of DNA demethylation specific to tTregs, with a relatively lower enrichment in aTregs. These observations indicate that autoimmune susceptibility SNPs exert a greater influence in tTreg development and function than in the misguided activation of autoreactive effector T cells. Furthermore, autoimmune SNPs are primarily associated with a loss of function in Treg cells, rather than a gain of function in Tconv cells, thereby predisposing the host to common autoimmune diseases.

Although functional defects in Tregs are detectable in the peripheral blood of T1D patients (56, 66, 67), their significance as indicators of Treg function within tissues is not well established. Studies in murine models, particularly in NOD mice, revealed that tissue microenvironments exacerbate specific Treg functional defects, which frequently evade detection in standard *in vitro* systems (68, 69). It is highly probably that a combination of factors, such as persistent inflammation, IL-2 pathway deficiencies, and limited TCR diversity, collectively undermine Treg functionality within the pancreatic islets (69–73). Consequently, optimal therapeutic strategies targeting regulatory T cells should aim to address this constellation of defects.

## 4 Tissue-specific adaptations, plasticity, and stability of Tregs

**Figure 2:**
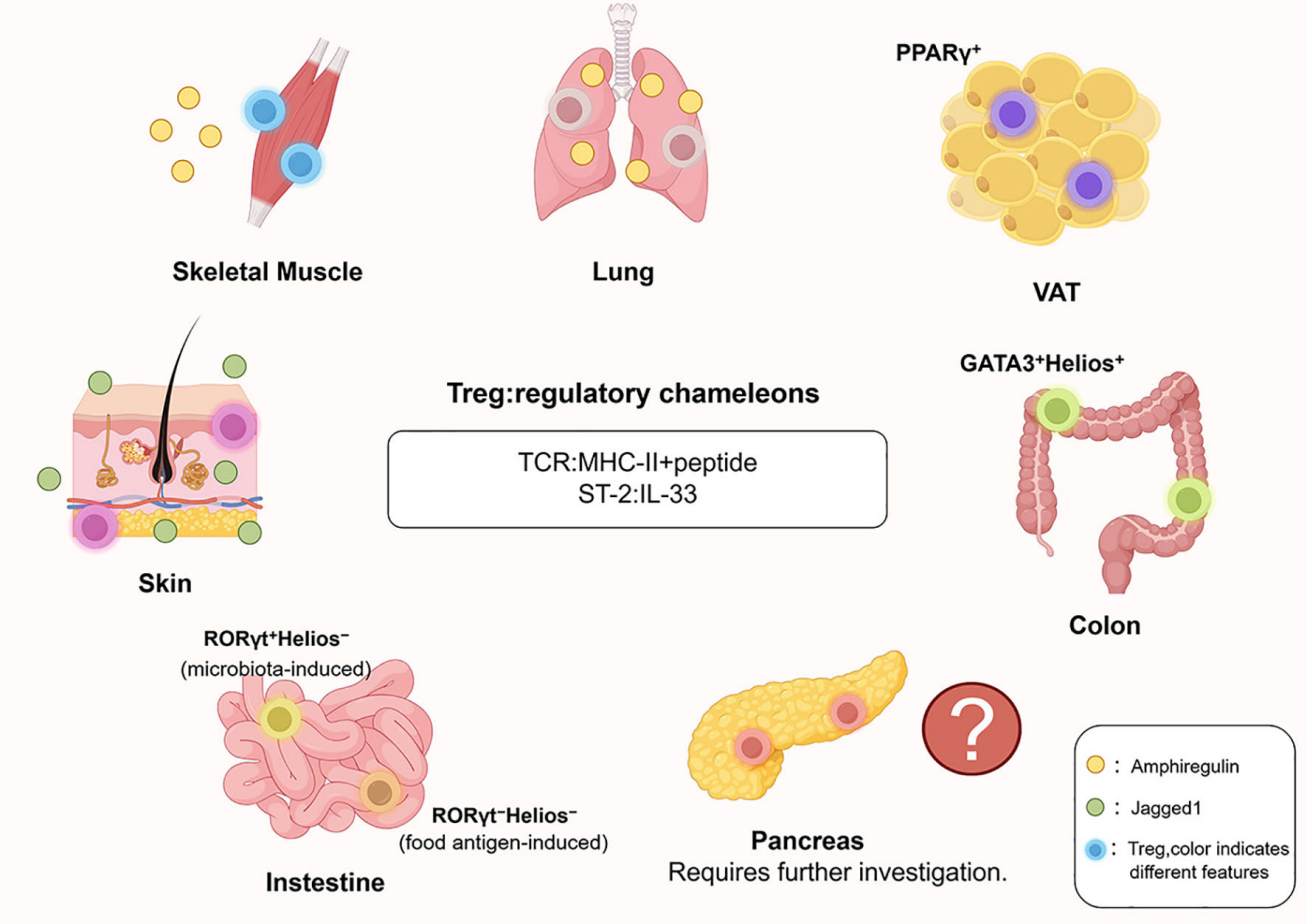
Tissue-resident regulatory T cells exhibit functional adaptability across non-lymphoid organs. This schematic illustrates the phenotypic and functional heterogeneity of tissue-resident Tregs (trTregs) in various non-lymphoid tissues. Beyond their classical immunosuppressive roles, trTregs contribute to tissue repair and metabolic homeostasis through context-specific mechanisms. Key examples include amphiregulin-producing Tregs in skeletal muscle and lung facilitating tissue regeneration; GATA3^+^Helios^+^ colonic Tregs responding to IL-33 to mitigate colitis; microbiota-induced RORγt^+^Helios^−^ and food antigen-induced RORγt^−^Helios^−^ intestinal Tregs maintaining immune tolerance; and PPARγ-expressing Tregs in visceral adipose tissue regulating inflammation and insulin sensitivity. Cutaneous Tregs support hair follicle regeneration via Jagged1, while the role of pancreatic Tregs remains under investigation. ST2–IL-33 signaling emerges as a unifying pathway for trTreg activation across tissues.

In addition to their function in preserving immune equilibrium within lymphoid organs, Tregs are also found in a variety of non-lymphoid tissues, where they exhibit significant functional adaptability (**Figure 2**). The functional diversity of these trTreg populations expands our traditional understanding of Tregs as generalized suppressors of inflammation. For instance, an increase in Treg frequency is observed in skeletal muscle after injury, a site where these cells are also found under healthy conditions. As expected, they suppress inflammation at this site, but also produce the growth factor amphiregulin, which enhances the regeneration of muscle satellite cells and promotes muscle repair (74). The tissue-repair function of Tregs is likewise vital in influenza-infected lungs; in this context, loss of amphiregulin production by Tregs in infected mice leads to increased damage to lung tissue (75). Visceral adipose tissue hosts a unique population of Tregs characterized by PPARγ expression, a transcription factor pivotal in adipocyte differentiation. This specific Treg subset serves to curb local inflammation, and its ablation impairs metabolic health, leading to deficits in insulin sensitivity (76, 77). Cutaneous trTregs are preferentially localized within the hair follicle stem cell niche of the bulge region (78). Within this niche, trTregs facilitate hair regeneration by secreting the Notch ligand Jagged1, which directly supports the proliferation and differentiation of local follicle stem cells. Moreover, diverse Treg subsets reside within the intestinal mucosa. One representative subset comprises GATA3^+^Helios^+^ colonic Tregs, presumed thymic in origin, rapidly sense the release of alarmin IL-33 during tissue damage, thereby orchestrating protection against colitis-associated immunopathology (79). The capacity to respond to IL-33 via ST2 expression emerges as a cardinal characteristic unifying multiple tissue-resident Treg populations, underscoring the importance of this canonical pathway for their function (75, 80, 81). By contrast, RORγt^+^Helios^-^ Tregs are peripherally induced in a process contingent on the gut flora. The ablation of RORγt expression in Tregs is sufficient to trigger severe intestinal inflammatory pathology (82, 83). Another distinct subset, RORγt^-^Helios^-^Tregs, concentrate in the upper small intestine, are primarily induced by food antigens and function to prevent allergic responses (84). These observations reveal the broad repertoire and functional versatility of Tregs, which safeguard tissue homeostasis by curbing inflammation and promoting repair processes. Remarkably, despite pancreas being the central target organ in T1D, the characteristics and functions of pancreatic Tregs have not yet been elucidated to the same depth.

A growing body of evidence points to an intimate connection between the functional stability of Tregs and their distinctive metabolic programming. Unlike pro-inflammatory Teffs (such as Th1 and Th17), which rely on glycolysis, Treg differentiation and functional maintenance require enhanced mitochondrial oxidative phosphorylation (OxPhos) and moderate levels of reactive oxygen species (ROS) signaling. Mitochondria-derived moderate ROS act as key signaling molecules, facilitating the expression of FoxP3, and the synthesis of anti-inflammatory cytokine IL-10, all of which are crucial for maintaining immune tolerance (85). Gerriets et al. noted that FOXP3 localizes to genes encoding subunits of PI3K and pyruvate dehydrogenase kinase PDK3, exerting a marked inhibitory effect; however, it remains incompletely understood how FoxP3 regulates the metabolic preferences of Treg cells (86). The most recent research indicates that dysregulated mitochondrial quality control profoundly impacts cellular identity and maturation of β-cells, brown adipocytes, and hepatocytes, through mechanisms such as mitochondrial-nuclear communication (87). Similarly, under conditions of dysregulated mitochondrial quality control, Tregs may also develop functional abnormalities. When mitochondrial DNA integrity, dynamics, or autophagic pathways are compromised, impaired OxPhos triggers the mitochondrial integrated stress response (ISR). This leads to alterations including chromatin remodeling, which subsequently promotes FoxP3 instability and functional impairment of Tregs. Collectively, these mitochondrial dysfunction-driven defects—propagating via stress-induced metabolite alterations to disrupt epigenetic regulation, which in turn impair Treg immunosuppressive capacity. Such functional erosion likely proves particularly consequential in autoimmune diseases with prominent metabolic dysregulation, exemplified by T1D, where it directly constrains Treg-mediated control of autoimmune responses.

The plasticity and stability of Tregs underpin their functional adaptability, yet the pathogenic risks associated with lineage instability warrant caution. When investigating Treg stability, it is crucial to distinguish between two distinct scenarios: lineage instability, in which Tregs completely lose their defining characteristics; and functional adaptability, whereby Tregs partially acquire certain features of effector T cells while retaining their core immunosuppressive function. As aforementioned, the expression of lineage-specifying transcription factor, FoxP3, is intimately linked to Treg stability. Experiments tracking the maintenance of FoxP3 using FoxP3 fate-reporter mice demonstrated that Tregs constitute a highly stable lineage under most conditions (88). However, instances of lineage instability can be observed, whereby former FoxP3^+^ cells downregulate this key transcription factor and adopt effector-like properties (89–91). It is plausible that a subset of FoxP3^+^ T cells only transiently expresses FoxP3 and lacks the complete epigenetic program characteristic of stable Tregs, potentially enabling their conversion into ex-Treg cells (34, 92).

Beyond complete lineage instability, Tregs also exhibit significant plasticity. Plasticity, defined as the ability to undergo functional conversion and differentiate into other cellular subsets (93), which is present in several CD4^+^ T cell populations, including T helper (Th) cells (94–98) and Tregs. Co-existing with conventional Th cells, a plastic Treg subset, termed Th-like Tregs, maintains fundamental Treg identity through FoxP3 expression while concurrently producing pro-inflammatory cytokines or expressing Th-associated transcription factors (99). The equilibrium between this hybrid population and Th cells is critical for immune homeostasis (100). Accumulating evidence indicates that Tregs can adopt features resembling those effector T cells, gaining the ability to express transcriptional regulators and chemokine receptors characteristic of Th1 (101, 102), Th17 (82, 83, 103), and T follicular helper (Tfh) lineages (104–106). Studies have also suggested the existence of Th2-like Tregs that express IRF4 and GATA3, although these DNA-binding proteins are likely involved in regulating a wide spectrum of effector-like Tregs, rather than being restricted to a phenotype that specifically mimics Th2 cells (35, 107, 108). Each Th-like Treg phenotype presumably confers the homing capacity to specific inflammatory sites to antagonize their cognate Teff populations (109).

Epigenetic factors and transcriptional regulatory networks serve as critical safeguards to maintain Treg stability and prevent their conversion into pathogenic phenotypes. Their function is to suppress the complete transformation of Tregs into pathogenic T cells, although they may not entirely prevent the acquisition of certain features, such as low-level production of inflammatory cytokines. For example, repressive histone modifications at the IL12RB2 promoter region dampen Treg sensitivity to IL-12, thereby limiting their entry into an aborted Th1 differentiation program. Within this program, although Tregs express T-bet and CXCR3, they produce only low levels of IFN-γ (101). Concurrently, the network of transcription factors within Treg cells actively suppresses the production of inflammatory cytokines. On the one hand, Tregs express the cytokine signaling suppressor SOCS1, which inhibits STAT1 and STAT3 signaling, thereby preventing robust production of IFN-γ and IL-17 (110). On the other hand, FoxP3 itself can directly bind to effector cytokine genes and repress their robust expression. Furthermore, the transcription factor Blimp-1 (B lymphocyte-induced maturation protein-1), essential for effector Treg function, attenuates IL-17 expression through its association with the IL17 locus, and promotes the repressive histone modifications (111). Collectively, these mechanisms explain why Th-like FoxP3^+^ cells and unstable Tregs are often associated with those changes.

The plasticity of Treg cells partially blurs the traditional distinction between pro-inflammatory and anti-inflammatory cellular lineages. Changes in Treg stability directly impact their suppressive function, reflecting the inherent complexity and adaptability of the immune system. In summary, functional adaptability acts as a double-edged sword in Treg biology. It is essential for directing Tregs to inflammatory sites, yet under specific conditions, this same adaptability can progress to lineage instability—marked by FoxP3 loss and conversion into potentially pathogenic ex-Tregs. The fundamental question of whether this phenomenon originates from preexisting heterogeneity within the Treg compartment—wherein a subset of cells is constitutionally prone to instability—or whether every Treg is capable of losing its identity when subjected to sufficiently strong inflammatory signals remains to be fully elucidated.

## 5 Identity and migration of pancreatic Tregs

### 5.1 Transcriptomic identity of pancreatic Tregs

As mentioned earlier, Tregs can be classified into three main subsets: tTregs, pTregs, and Tr1. This is evidence that all three populations are able to suppress islet autoimmunity (58). However, the absence of definitive markers to discriminate tTregs from pTregs hampers the assessment of their individual contributions to T1D pathogenesis. Therefore, delineating the identity markers of Tregs is crucial for determining the origin(s) of reactive Treg populations, which warrants the elucidation of the allelic susceptibility to Treg dysfunction and the upstream immune defects in T1D setting (27).

The progression and remission of T1D involve specific Treg subsets and their distinct transcriptomic profiles. Benoist and colleagues conducted studies to compare the transcriptomes between pancreatic and splenic Tregs in NOD mice, and identified that the pancreatic Tregs are featured by the upregulation of suppressive molecules (such as IL10 and LAG3), specific chemokine receptors (CXCR3 and CCR5), and activation-associated transcription factors (Nr4a2, Fos, and Jun) (112). Beyond this shared effector profile, a signature more specific to pancreatic Tregs is enriched in genes governing cell growth and proliferation, suggesting a higher local turnover rate (69, 113). Additionally, this unique identity is further underscored by a lack of typical tissue-resident transcription factors (112). Subsequent analysis revealed that genes associated with Treg activation signatures are significantly upregulated in the pancreatic Treg transcriptome. Furthermore, pancreatic Tregs also displayed a bias toward the BLIMP-1 and IRF4-dependent Treg transcriptomic signatures; these transcription factors are critical for Treg effector functions and their maintenance within tissues (35, 107). However, the most pronounced differences were observed in the expression profile of CXCR3^+^ Tregs. The induction of CXCR3 depends on the expression of the transcription factor T-bet in Tregs and other leukocyte subsets (9, 24, 37). CXCR3 and T-bet profoundly influence the dynamic migration of Tregs (to be discussed in detail later). Collectively, these results indicate that Treg cells located at sites of autoimmune responses possess a specific identity characterized by enhanced effector function, accelerated cell turnover, and a prominent CXCR3^+^ Treg signature program, which may bolster their capacity to control type 1 inflammatory responses within the islets.

### 5.2 Single-cell insights into Treg heterogeneity

Emerging technologies have substantially advanced the classification of Treg subsets. In humans, Mass cytometry using 26 well-established Treg-associated markers has resolved 22 distinct subsets (114), while transcriptomic analyses have corroborated their differentiation into Th-like lineages and defined subset-specific molecular signatures for both Th cells and Tregs (115). However, none of these classification schemes rely on unsupervised global gene expression profiling. Recent advances in single-cell RNA sequencing (scRNA-seq) have provided novel insights into T cell and Treg heterogeneity at single-cell resolution (116–120). Using scRNA-seq data, Treg cells were subdivided into 6 clusters in healthy human peripheral blood and 5 clusters within the human breast cancer microenvironment (116). Despite these findings, in-depth single-cell studies of human Treg cells under steady-state or disease conditions remain limited. The identity, functional/steady-state characteristics, differentiation pathways, and interrelationships of distinct Treg subsets are still incompletely defined. scRNA-seq has revealed significant dynamic shifts in immune cells at different stages of T1D, particularly during partial remission. The study identified marked fluctuations in the proportions of TIGIT^+^CCR7^−^ Tregs and CD226^+^CCR7^−^CD8^+^ T cells and validated, using machine learning based algorithms, that these two subsets serve as biomarkers for declining β-cell function (121). Treg heterogeneity in humans has been further resolved by single-cell transcriptomic analysis. In particular, eTregs can be subdivided into a FOXP3^hi^ subset with potent suppressive function and a highly proliferative MKI67^hi^ subset, developing along two distinct differentiation trajectories (Path I/II) (21).Although these findings are primarily based on peripheral blood data, they still provide crucial insights for understanding the features of pancreas trTregs.

Recent studies that integrate single-cell transcriptomic data from humans and mice have systematically elucidated the conserved features and species-specific adaptations of Treg cells throughout evolution. In terms of conservation, Treg cells in both species express core transcriptional markers such as FOXP3 and IKZF2, which allow for a clear distinction from Tconv cells. Additionally, both species possess a “furtive” Treg subset characterized by low suppressive activity (119). Furthermore, Treg cells exhibit transcriptional dynamics along a tissue-adaptation continuum and display conserved expression programs between homeostasis and disease, as well as between mice and humans (117). However, notable interspecies molecular differences exist within this conserved regulatory framework. Certain signaling molecules demonstrate paralogous gene substitution; for example, Pim1 in mice is analogous to Pim2 in humans. Moreover, the expression profiles of some tissue-homing receptors and effector molecules reveal species-specific preferences (117). These findings indicate that although Treg adaptation is conserved among species, its molecular mechanisms exhibit evolutionary plasticity. This has significant implications: the conserved features support the use of mouse models to investigate fundamental aspects of human Treg biology, whereas species-specific differences require the validation of human therapeutic targets. Future integration of cross-species single-cell data will facilitate the identification of conserved pathways and distinct human characteristics, thereby advancing precision immunotherapy.

### 5.3 Dynamic trafficking behavior of pancreatic Tregs

The homing and localization of pancreatic Tregs depend on a complex chemokine-receptor network, and their dynamic migration patterns are critical for local immune suppression. Notably, pancreas trTregs often exhibit islet antigen specificity, which is closely linked to their dynamic migratory behavior. The accumulation pattern of Tregs within the pancreas, particularly in the islets, is finely regulated by adhesion molecules, chemokines, and their receptors, whose expression is influenced by both Treg-intrinsic factors and the pancreatic microenvironment. Importantly, emerging evidence highlights that endothelial cells (ECs), comprising both blood (BECs) and lymphatic (LECs) endothelial cells, are integral and active components of the tissue microenvironment, serving as essential gatekeepers of Treg trafficking and localization (122). Through bidirectional crosstalk involving molecules such as LTα1β2–LTβR, PD1–PDL1, and S1P–S1PRs, endothelial cells not only facilitate Treg transmigration but also modulate their stability, differentiation, and immunosuppressive function (122). In the context of T1D, the accumulation of CXCR3^+^ Tregs within the islets is impaired. This phenomenon has significant functional consequences, as evidenced by that CXCR3-deficient mice fail to effectively suppress Th1 activity and develop diabetes earlier (123). The deficit caused by impaired CXCR3 signaling is partly counteracted by the activity of additional chemokine receptors, notably CCR2, CCR8, and CXCR6, which are also present on pancreatic Tregs and support their accumulation within islets. Pioneering work on Treg trafficking in transplanted islets demonstrated that inflamed islets provide an essential “educational” environment for Tregs prior to their migration to the draining lymph nodes (124). Differential usage of homing mechanisms guides Tregs to distinct sites: migration to the inflamed islets implicates CCR2, CCR4, CCR5, and P-/E-selectin ligands, whereas recruitment to the draining lymph nodes employs CCR2, CCR5, and CCR7 (124). Furthermore, Treg positioning is dependent on islet-derived factors (e.g., CCL12, MAdCAM-1) and Treg-expressed integrins (α4 and β7) (125–128). Pancreas-resident APCs, particularly F4/80^+^ macrophages and CD11c^+^ dendritic cells, are a primary source of CXCL9, CXCL10, and CXCL11. These ligands mediate the specific recruitment of CXCR3-expressing Tregs into the pancreatic islets (129). This empirical evidence underscores that the functional efficacy of tissue Tregs is inextricably linked to their migratory capacity, which is enabled by a series of factors operating with substantial redundancy. This functional compensation, however, raises a central unanswered question: what is the hierarchical contribution of these various molecules to Treg migration specifically between the pancreatic parenchyma and the pancreatic lymph nodes (PLN). Similarly, it remains unclear whether these migration patterns executed by pancreatic Tregs are primarily associated with T1D or represent a general phenomenon occurring in other inflammatory responses. Further investigation into the endothelial-Treg crosstalk within the pancreas may unveil novel targets for restoring immune balance in T1D and beyond.

### 5.4 The dual role of T-bet in Treg migration and function

The expression of transcription factors also influences the migration of Treg cells in T1D, with T-bet exhibiting the most pronounced effect. However, its function is context-dependent and cannot be simply categorized as either detrimental or beneficial to Treg suppressive capacity. On the one hand, T-bet plays a guiding role in Tregs by promoting their migration to inflammatory sites (130, 131). For instance, it upregulates CXCR3 expression, facilitating Treg recruitment to Th1-inflammatory regions such as pancreatic islet, where CXCL9 and CXCL10 are enriched (131, 132). Moreover, T-bet-specific knockout in Tregs inhibits IFN-γ production and impairs their ability to suppress Th1 effector cells (102, 130). Some studies even suggest that T-bet^+^ Tregs may specialize in suppressing effector T cells that also express T-bet (102). On the other hand, multiple studies indicate that T-bet deficiency does not impair, and may even enhance, the overall suppressive function of Tregs (131). T-bet-deficient Tregs have been shown to suppress effector T cells as effectively as or more effectively than wild-type Tregs, both *in vitro* and *in vivo* (133–135). This apparent paradox, where functional maintenance or enhancement coexists with altered migratory behavior, can be explained by multiple migration and retention mechanisms regulated by T-bet. Specifically, although loss of T-bet impairs CXCR3-dependent migration to the pancreatic islets, T-bet^−^/^−^ Tregs upregulate CCR4, enabling compensatory migration and accumulation in the pancreas via CCL22-mediated signaling (124, 136). Concurrently, T-bet deficiency upregulates CD103 (αE integrin), which binds to E-cadherin and promotes anchorage of Tregs within epithelial tissues, thereby enhancing retention of T-bet^−^/^−^ Tregs in the islets (132, 137–139). Moreover, T-bet regulates S1PR1 expression, and its deficiency leads to reduced S1PR1 levels, which impairs Treg egress from pancreatic islets back into the bloodstream, thereby further promoting Treg local retention (140, 141). Collectively, in the context of T1D, T-bet does not act as a functional “on/off switch”, rather as a “navigation commander” for Tregs. Its absence does not fundamentally disrupt Treg suppressive capacity but instead reprograms their migratory behavior and tissue-localization properties. T-bet^−^/^−^ Tregs tend to be recruited via alternative pathways and retained longer within target tissues. In fact, this is consistent with the concept that T-bet^+^ Tregs differentiate from T-bet^−^ precursors (102). In conclusion, the impact of T-bet deficiency in Tregs should not be simply interpreted in binary terms of benefit or harm, rather its essential role lies in regulating the spatiotemporal distribution and migratory dynamics of Tregs. These effects may yield diverse consequences in the complex immune landscape of T1D, and understanding how T-bet fine-tunes the tissue-specific localization of Tregs is critical for evaluating its potential as a therapeutic target.

Importantly, the fundamental mechanisms governing Treg migration are not unique but are common to pathogenic effector cells. As illustrated in NOD mice, high α4β7 expression on islet-infiltrating lymphocytes indicates that MAdCAM-1 within the inflamed islet microenvironment also mediates the recruitment of pathogenic Teffs, implying a common homing pathway (128, 142). During T1D progression, a mixed population of immune cells including lymphocytes with both naïve and memory phenotypes, dynamically and continuously infiltrates into the pancreatic islets to mediate β cell destruction. It is worth noting that the active migration of Tregs into these insulitic lesions is lower compared to their conventional CD4^+^ counterparts (143). Therefore, it would be interesting to dissect when and how preferential recruitment of Tregs into the pancreatic islet occurs. A more comprehensive understanding of the detailed migration mechanisms required for Treg accumulation within the pancreas remains to be elucidated. This knowledge is of great importance for the expansion and development of therapeutic Tregs capable of responding to tissue-specific environmental cues.

## 6 Immunotherapeutic breakthroughs targeting Tregs in T1D

Autoimmune diseases (ADs), such as T1D, constitute a global health burden impacting millions of subjects, a problem further compounded by their consistently rising prevalence. At present, the absence of curative interventions remains a challenge in the management of autoimmune diseases. Available therapies primarily focus on symptom management and often involve immunosuppressive agents, which can be associated with significant adverse effects. As a consequence, scientists have been dedicated to developing safe and effective targeted therapies for the past few decades (**Table 2**).

**Table 2:** Comparison of major Treg immunotherapies for T1D.

Therapeutic approach	Advantages	Major challenges
Autoantigen Vaccine	• High antigen specificity • Excellent safety profile; avoids broad immunosuppression • Potential for disease prevention in at-risk individuals	• Modest clinical efficacy • Low bioavailability (e.g., oral degradation) • Efficacy limited to specific patient subgroups
Low-dose IL-2 Therapy	• Effectively expands endogenous Tregs • Directly enhances Treg function and survival	• Narrow therapeutic window • Off-target activation of effector cells (e.g., eosinophils) • Transient effects; no sustained efficacy in established disease
Adoptive Polyclonal Treg Transfer	• Proven clinical safety in trials • Achieves sustained C-peptide preservation in a subset of patients	• Variable and often limited long-term persistence • Modest overall efficacy; lacks antigen specificity • Scalable GMP manufacturing is complex and costly
Genetically Engineered Tregs (EngTregs)	• High precision via antigen-specific (TCR/CAR) targeting • Potent suppressive capacity with bystander effect • Function and persistence can be enhanced via genetic design	• Highly complex and costly manufacturing • Long-term safety risks (e.g., insertional mutagenesis) • Optimal target antigen and TCR affinity remain unclear
Combination/Integrated Strategies (e.g., GNTI-122)	• Synergistic design addresses multiple limitations simultaneously (stability, targeting, survival) • Incorporates safety switches (e.g., CISC) for controllable activation	• Most complex development and regulatory pathway • Not yet in clinical testing; efficacy and safety unproven in humans

### 6.1 Vaccine therapy: inducing antigen-specific tolerance

Insulin is the most abundant protein in pancreatic β cells. Insulin autoantibodies (IAAs) are typically the first to appear during the pathogenesis of T1D, highlighting the pivotal role of insulin in initiating T1D autoimmunity (144). Therefore, inducing insulin-specific Tregs holds promise for effectively controlling autoimmunity. T1D vaccine therapy, also known as autoantigen-specific immunotherapy, aims to restore immune tolerance and induce antigen-specific Tregs by targeted delivery of autoantigens (e.g., insulin, GAD65). Its core mechanism involves the processing of subimmunogenic doses of antigens (e.g., insulin mimotope peptides) by APCs, which subsequently activate islet antigen-specific CD4^+^ T cells to differentiate into FoxP3^+^ Tregs, rather than Teffs. These Tregs exhibit high expression of CTLA-4, IL-2Rα, and TIGIT, and they precisely regulate autoimmune responses by suppressing Teff activity and promoting the expression of immune tolerance-associated genes (e.g., FoxP3, IL-10) (145). For instance, in an HLA-DQ8 transgenic humanized mouse model, an insulin vaccine successfully induced stable and functional Treg clones that effectively suppressed β-cell destruction (145). The key advantages of this approach lie in its antigen specificity, safety profile, and preventive potential. Vaccines can precisely target pathogenic autoimmune pathways, circumventing broad immunosuppression, and thereby minimizing disruption to normal immune function (145). Furthermore, the use of subimmunogenic vaccine doses avoids triggering Teffs activation, significantly reducing the risk of adverse effects (145). Vaccine therapy is also applicable to high-risk individuals, such as autoantibody-positive relatives, and has the potential to delay or even prevent the onset of certain diseases like T1D (146). However, vaccine therapy may manifest several limitations. First, its efficacy has been modest. For example, oral insulin failed to effectively preserve residual β-cell function in patients with new-onset T1D and did not markedly delay the progression of disease in at-risk populations with pre-diabetes (146, 147). Second, vaccines often suffer from low bioavailability, and oral administration is particularly susceptible to gastrointestinal degradation, leading to insufficient antigen presentation efficiency (147). Additionally, patient heterogeneity poses a significant challenge, as vaccine efficacy may be restricted to specific subgroups (e.g., those with high IAA levels), necessitating precise stratification for optimal therapeutic outcomes (146). Therefore, the clinical application of antigen-specific Treg therapy continues to face obstacles in the context of autoimmune diseases. Moreover, the ability to directly identify and characterize human insulin-specific FoxP3^+^ Tregs *in vitro* is crucial for assessing responses to insulin-specific vaccination.

### 6.2 IL-2 therapy: expanding and activating endogenous Tregs

To overcome vaccine limitations, IL-2 therapy exploits Treg high-affinity IL-2R dependency to expand and enhance their function. Tregs exhibit heightened sensitivity to fluctuations in IL-2 concentration due to their expression of the high-affinity IL-2R (44, 148). Administration of IL-2 has been demonstrated as an effective strategy to induce *in vivo* Treg expansion and prevent autoimmunity in numerous mouse models, such as in NOD mice (149). However, IL-2 exhibits dual anti-inflammatory and pro-inflammatory effects, as it can also non-specifically expand pro-inflammatory immune subsets. This differential cellular response depends on the hierarchical expression levels of IL-2R across distinct lymphocyte subsets (150). Under normal physiological conditions, IL-2 produced by activated Teffs expands and sustains the Treg population, while Tregs reciprocally maintain immune homeostasis by feedback-inhibiting Teff responses. Disruption of this reciprocal interaction leads to dysregulation of the Treg/Teff ratio and promotes autoimmune responses in NOD mice. Notably, within the context of T1D, Tregs residing in the pancreas exhibit greater sensitivity to IL-2 compared to Tregs in the PLNs and other sites (69). IL-2 treatment increases the proportion of Tregs in the pancreas of prediabetic mice; however, in mice with new-onset diabetes, where pancreatic Treg proportions are already markedly elevated, IL-2 therapy fails to further augment their numbers (69). Studies have excluded proliferation as the primary mechanism underlying IL-2-mediated increases in Treg numbers. Furthermore, IL-2 signaling promotes Treg survival (69), recruitment, and synergizes with TGF-β to induce the conversion of CD4^+^ T cells into Tregs (151). Beyond increasing pancreatic Treg numbers, IL-2 also directly enhances the activity of pancreatic Tregs by upregulating the expression of molecules critical for Treg function including CD25, FoxP3, CTLA-4, ICOS, and GITR (152), coupled with immunosuppression within the islets during T1D progression. It is therefore critical to elucidate the mechanisms through which IL-2 can selectively engage Tregs, particularly those localized in the pancreatic islets. To this end, Mark and colleagues engineered a β-cell-targeted IL-2 delivery system using an adeno-associated viral (AAV) vector to direct localize the expression of IL-2 within the islets of NOD mice (153). Consistent with systemic IL-2 therapy, β-cell-specific IL-2 delivery persistently suppressed β-cell autoimmunity and provided long-term protection against diabetes onset in both preclinical and advanced disease stages. However, it showed no therapeutic efficacy in NOD mice with new-onset diabetes (153). Furthermore, this approach failed to induce sustained proliferation of islet-resident Tregs (153). Functionally, pancreatic Tregs converted to a CD62L^high^Foxp3^+^ Treg phenotype, exhibiting enhanced fitness and suppressive capacity. Notably, this effect was not associated with increased levels of IL-10 or TGF-β (153). Although β-cell-specific IL-2 demonstrated efficacy comparable to systemic IL-2 in preventing T1D, these data suggest that a β-cell-targeted strategy may exhibit advantage for effectively sustaining the functional capacity of intra-islet Tregs within the context of T1D.

Of note, several alternative strategies have been developed to achieve more specific targeting of Tregs. For example, the use of specific anti-IL-2 monoclonal antibodies (mAbs) to block the IL-2 binding site on the β-chain (CD122) enables the formation of IL-2/antibody complexes that preferentially target Tregs. This selectivity stems from the fact that Tregs, unlike most other immune cells, primarily rely on CD25 for IL-2 binding. These engineered IL-2 complexes exhibit the ability to preferentially expand both murine and human Tregs *in vitro*, representing a promising approach for future targeted immunomodulatory therapies (154). Another strategy involves engineered IL-2 muteins, which are designed with reduced affinity for CD122 to increase CD25 dependency (150). However, even with enhanced selectivity, CD25 can be expressed on a range of immune cells beyond Tregs, potentially leading to off-target effects and associated adverse events (155).

### 6.3 Adoptive Treg transfer: restoring tolerance via cell infusion

Given the limitations of endogenous immunomodulatory therapies, such as IL-2 therapy, the adoptive transfer of exogenous Tregs offers a promising new approach for treating T1D. This innovative immunomodulatory strategy involves isolating immunosuppressive CD4^+^CD127^low^CD25^+^ Tregs from the patient’s own peripheral blood, followed by large-scale ex vivo expansion and subsequent reinfusion into the patient (156). Its primary objectives are to restore immune tolerance and suppress the autoimmune attack by Teffs on pancreatic β cells, thereby delaying disease progression. Significant progress has been made in this therapy. Technically, a GMP-compliant expansion protocol utilizing artificial APCs, such as the KT64/86 system, has been successfully established. This protocol achieves over 3000-fold expansion of Tregs while maintaining high FoxP3^+^ expression and robust suppressive function (157, 158). Clinically, despite concerns regarding Treg lineage instability raised previously, Phase I trials confirmed that a majority of infused CD25^+^CD127^low^ Tregs expanded ex vivo from autologous polyclonal sources, maintain phenotypic fidelity in T1D patients. These expanded Tregs demonstrate favorable safety (no serious adverse events) and durable persistence, with ~25% of peak cell levels detectable at 1-year post-infusion. Critically, sustained C-peptide preservation beyond two years in 38% of recipients provides compelling rationale for advancing to Phase II efficacy trials (156).

For Treg cell isolation, cells enriched by fluorescence-activated cell sorting (FACS) in Fr. I represent a more suitable starting population for expansion. This preference stems from the ability to avoid potential contamination by non-Treg cells present in Fr. III (159), although the Fr. I population itself is not entirely homogeneous. Current mainstream methods commonly use extensive polyclonal or antigen-driven expansion to propagate Tregs in an undifferentiated state. A potential alternative strategy involves purifying specific functional Treg subsets, such as follicular regulatory T cells or CXCR3^+^ Th1-like Treg cells, to expand them ex vivo. This approach aims to more precisely modulate the autoimmune responses mediated by Tfh or Th1 cells, respectively. However, the efficacy of this targeted strategy remains to be fully validated. Furthermore, it is still unclear whether its benefits outweigh those of maintaining Tregs in an undifferentiated state, an approach that preserves their functional plasticity and enables them to respond to a diverse array of signals following adoptive transfer *in vivo*.

### 6.4 Engineered Tregs: precision engineering for enhanced potency

Although the safety of autologous Treg adoptive transfer has been established, its therapeutic efficacy remains constrained by factors such as antigen non-specificity, low *in vivo* persistence, and insufficient cell numbers. While the overall efficacy of polyclonal Treg therapy has been modest, the observation of long-lived Tregs underscores the need to develop novel strategies to enhance Treg functionality, survival, and proliferative capacity within the host (156, 160). In recent years, genetically engineered Tregs (EngTregs) has emerged as an innovative solution for precision therapy in T1D. Compared to biologics and small-molecule drugs, living cells, particularly immune cells, can sense combinatorial environmental signals and orchestrate sophisticated, controllable therapeutic responses accordingly. In other words, molecular drugs function akin to single-task tools, whereas cells resemble programmable devices capable of deploying context-appropriate combinations of tools based on the specific situation. Consequently, engineering immune cells, such as EngTregs, holds promise for achieving smarter and more dynamic immunomodulation.

#### 6.4.1 Engineering Treg stability

Currently reported EngTregs are typically not derived directly from natural Tregs, but are generated through genetic engineering of CD4^+^ T cells. The adoption of this strategy stems from limitations in isolating primary Tregs. While Tregs can be enriched from peripheral blood using combinatorial surface markers (e.g., CD4^+^, CD25^high^, and CD127^low^) (8), this approach is constrained by insufficient cell yields and the risk of contamination by conventional T cells (which may also express CD25 under certain conditions). Although stimulation with TGF-β combined with TCR engagement can transiently induce FOXP3 expression and confer suppressive function in CD4^+^ T cells, this induction is unstable and reverses upon withdrawal of stimuli (161). As a result, a strategy involving viral vector-mediated overexpression of the FOXP3 gene (e.g., using retroviral or lentiviral vectors) is preferred. This method successfully induces stable expression of Treg signature markers (e.g., CD25, CTLA-4), suppression of pro-inflammatory cytokine production, and acquisition of potent suppressive function in murine (12, 29) and human (162, 163) CD4^+^ T cells *in vitro*. Significantly, FoxP3-transduction-based cell therapy has advanced to clinical translation. A first-in-human clinical trial targeting patients with IPEX syndrome is currently actively recruiting participants. This trial employs a lentiviral vector to deliver a functional FOXP3 gene into patient-derived autologous T cells, followed by reinfusion therapy (NCT05241444) (164). This represents a significant milestone in transitioning the technology from proof-of-concept to clinical application. To further enhance safety and optimize expression control, recent studies have adopted more precise gene editing strategies. For instance, leveraging recombinase-mediated homology-directed repair (HDR) to site-specifically integrate a strong promoter sequence (e.g., the engineered MND promoter: myeloproliferative sarcoma virus enhancer, with deletion of the negative control region and substitution of the dl587rev primer binding site) upstream of the endogenous FOXP3 locus (165). Compared to the random integration associated with viral vectors, this approach significantly reduces the risks of genotoxicity, gene silencing, and expression variability, thereby offering a novel pathway to achieve physiological and stable regulation of FoxP3.

#### 6.4.2 Conferring antigen specificity: TCR-Tregs versus CAR-Tregs

Once sufficient EngTregs are generated, the next key issue is to enhance their target specificity. TCR transduction technology represents a core strategy to overcome this hurdle. Given that T1D features well-defined autoantigen targets, this field presents a unique opportunity for TCR-engineered Tregs (TCR-Tregs). Crucially, researchers have successfully isolated multiple TCR clones from both islet-infiltrating pathogenic T cells and Tregs (166). These TCRs target epitopes of diverse T1D-associated autoantigens, including GAD65, G6PC2(also known as IGRP, Islet-specific Glucose-6-phosphatase catalytic subunit-Related Protein), Preproinsulin (PPI), Proinsulin or insulin (166) and a growing repertoire of TCRs specific for Hybrid Insulin Peptides (HIPs) (167, 168). Integrating TCR gene transduction with FoxP3 genetic engineering enables the reprogramming of human or murine T cells into antigen-specific EngTregs, which then target the defined T1D-relevant peptides (e.g., human G6PC2, GAD65, or PPI, or murine 2.5HIP), endowing them with potent suppressive function. For instance, Buckner’s team employed HDR for FOXP3 integration coupled with TCR transduction to construct islet-specific engineered Tregs. These engineered Tregs exhibited antigen specificity and demonstrated bystander inhibition, a phenomenon where Tregs suppress Teffs driven by a specific antigen, and concurrently suppress Teffs recognizing distinct antigens. This suppressive effect extends beyond Teffs directly interacting with Tregs to neighboring Teffs, even without direct Treg-Teff contact (169).

A pivotal determinant of TCR-Treg therapeutic efficacy is the affinity of the TCR for its target pMHC or the overall binding strength (avidity). It remains controversial whether low-affinity or high-affinity TCRs are coupled with higher advantage for Treg therapeutic potency. Several lines of evidence support a link between high TCR affinity and enhanced suppressive function. First, Treg development is thought to arise from agonist selection rather than negative selection. During negative selection, widespread expression of self-antigens in the thymus leads to clonal deletion. In contrast, tissue-restricted antigens mosaically expressed by medullary thymic epithelial cells generate intermittent, suboptimal TCR signals that drive the differentiation of naïve T cells into the regulatory lineage. This explains why thymic Tregs exhibit a TCR repertoire strongly biased toward self-antigens from specific peripheral tissues, suggesting that high-affinity TCRs may confer functional superiority (170). Furthermore, in transplantation models, Tregs expressing TCRs with higher functional affinity demonstrated stronger suppression *in vitro* and prolonged allograft survival *in vivo* compared to their low-affinity counterparts (171). Similarly, in human T1D settings, engineered Tregs expressing a high-affinity TCR specific for GAD555–567 (clone R164) suppressed GAD-reactive effector T cells more effectively than those expressing a lower-affinity TCR (clone 4.13) against the same epitope (172). Collectively, higher affinity may enable Tregs to effectively compete for limited antigen and achieve robust activation within inflammatory niches. Conversely, compelling evidence supports the sufficiency and potential advantages of low-affinity TCRs. Engineered Tregs expressing TCRs derived from islet-infiltrating conventional T cells from patients potently prevented disease. These Tregs homed to the pancreas and mediated antigen-specific and bystander suppression. This finding is significant because it indicates that even TCRs from autoreactive effector T cells—typically possessing low-to-medium affinity due to thymic selection—can program Tregs to exert potent suppression (169). Interestingly, work by Maria Bettini’s group revealed that both high- and low-affinity Tregs can be recruited to the pancreas and contribute to protection, albeit potentially through distinct mechanisms: high-affinity Tregs preferentially upregulate TCR-dependent mediators such as IL-10 and CTLA-4, whereas low-affinity Tregs express higher levels of tissue repair factors like amphiregulin (173). More recently, they further demonstrated that the suppressive capacity of Tregs correlates with the TCR affinity of the Teffs they target: Tregs with low-affinity TCRs effectively suppressed islet infiltration by Teffs of similarly low affinity but were less able to control high-affinity Teffs (174). This underscores that the functional balance between Tregs and Teffs may be more critical than TCR properties alone in determining autoimmune outcomes. Additionally, Tregs possess an intrinsic capacity to amplify TCR signaling independent of affinity. Even when expressing identical TCRs as Teffs, Tregs convert TCR engagement into stronger downstream signaling. However, under intense inflammatory stress, this intrinsic signaling advantage may be insufficient to fully compensate for lower TCR function, thereby contributing to T1D pathogenesis (174). This offers an important insight for future antigen-specific Treg therapies: successful strategies may not depend solely on selecting the highest-affinity TCRs but should also focus on enhancing or mimicking Tregs’ inherent signal-amplifying potential to improve their functional stability in high-inflammatory environments.

When designing TCR-Tregs for T1D treatment, the source and specificity of the TCR are critical. Given that Teffs associated with T1D are more abundant than Tregs within islet infiltrates, they constitute a broader repository of potentially effective TCRs. Therefore, current research predominantly utilizes TCRs derived from islet-reactive conventional CD4^+^ T cells (169, 172, 174, 175). In contrast, TCRs originating from islet-infiltrating Tregs have been relatively understudied (174, 176) and no comprehensive comparison has been conducted on the efficacy of Treg-derived versus conventional T cell-derived TCRs.

Although TCR-Treg products have not yet entered into clinical trials, T1D could be a highly suitable autoimmune disorder for this therapy due to its well-defined repertoire of disease-associated autoantigens and strong association with specific HLA haplotypes. Of note, the therapeutic advantage of targeting certain antigens (e.g., hybrid peptides) remains unclear, primarily due to the complete absence of comparative studies evaluating different TCR-antigen pairs *in vivo*. To bridge this gap, Abata Therapeutics has introduced ABA-201, an investigational therapy consisting of T1D-specific TCR-engineered Tregs (177). Clinical trials are scheduled to initiate in 2025. Yet, critical challenges regarding TCR selection remain unresolved in the field, such as the choice of antigen target, origin of the receptor, binding strength and specificity, as well as HLA restriction.

An alternative approach to confer antigen specificity to Tregs is through the chimeric antigen receptors (CARs). A typical CAR structure consists of an extracellular antigen-binding domain, derived from a single-chain variable fragment (scFv) of an antibody, linked via a hinge and transmembrane domain to an intracellular signaling domain that triggers T cell activation (178). The distinction between CAR T cells and CAR Tregs lies in their applications: CAR T cells are primarily used in malignancies to directly recognize tumor-specific antigens and eliminate cancer cells, while CAR Tregs are employed in ADs to suppress autoreactive immune responses. Unlike TCRs, CARs are not restricted by MHC presentation, offering greater flexibility for application across patient populations with variable HLA haplotypes. CARs are also characterized by a modular architecture that allows selective configuration of individual components to attain specific functional properties (179). A key constraint, however, is their dependence on cell membrane-associated or oligomeric antigens to facilitate receptor crosslinking, which restricts their application against intracellular proteins or secreted soluble monomers (178). Early generated CAR constructs are typically featured by a solitary CD3ζ signaling module, while follow up designs have integrated additional co-stimulatory domains, such as those from CD28 or 4-1BB, to augment functional potency (178). Most CAR-Treg therapies presently under development combine CD28-based co-stimulation with the core CD3ζ signal (178). Nevertheless, systematic comparisons of co-stimulatory domains remain relatively limited (179), and novel CAR architectures enabling cytokine signaling, safety switches, or response logic gating continue to emerge (180). CAR technology remains relatively unexplored in the context of T1D therapy due to challenges in identifying optimal targets. However, alternative strategies aimed at peptide–MHC complexes, HLA mismatches in islet transplants, or inflammation-related markers represent viable alternatives. Generally, TCR-based therapies benefit from the relative feasibility of isolating TCR sequences from insulin-infiltrating T cells. In contrast, CARs, if designed against relevant antigens, could offer greater flexibility to fine-tune Treg function and tissue specificity. Overall, TCR- and CAR-engineered Tregs possess distinct advantages and limitations (**Table 3**) (181, 182).

**Table 3:** Key feature comparison — TCR-Tregs and CAR-Tregs.

Feature	CAR-Tregs	TCR-Tregs
Antigen Recognition	Recognize surface antigens via an scFv; MHC-unrestricted.	Recognize peptide-MHC complexes via the natural TCR; MHC-restricted.
Antigen Scope	Limited to cell surface and oligomeric antigens.	Capable of recognizing both intracellular and cell surface antigens (via MHC presentation).
Stability & Persistence	Stability is variable and may be compromised by excessive artificial signaling; evidence for *in vivo* persistence is still accumulating.	Exhibit high stability; evidence from TCR-T cells suggests they can persist long-term *in vivo* and provide sustained immune surveillance
Customizability	Highly customizable: Flexible selection of targets, signaling domains, etc.; less dependent on IL-2.	Limited to the natural TCR repertoire; lower flexibility for customization.
Functional Risks	Risk of dysfunction or impaired survival; may adopt a strong effector-like phenotype.	Function is more physiological; however, risks include inadequate activation or suboptimal suppression.

#### 6.4.3 Advances in genome editing and delivery platforms

The CRISPR–Cas system, due to its simplicity, high efficiency, and robustness, has become a central tool for the genetic engineering of Tregs. This system encompasses various types, with the most widely used being the Class 2 Type II Cas9 protein (183). It enables precise targeting of specific genomic loci through easily designed guide RNAs (gRNAs) and facilitates gene knockout, knock-in, and even base editing via non-homologous end joining (NHEJ) or HDR mechanisms (183), significantly surpassing earlier technologies such as meganucleases and zinc-finger nucleases in both efficiency and programmability (184).

Since the initial demonstration that primary human T cells could be genetically edited using CRISPR-Cas9, substantial progress has been made in executing CRISPR-mediated genetic modifications in human T cells (185). Achievements include biallelic knockout with efficiencies exceeding 80% (186) and the precise knock-in of multiple genes at defined genomic loci in primary human T cells (187). This progress has been facilitated, in part, by advancements in delivery methods. In the evolution of delivery technologies for T-cell engineering, lentiviruses were among the first FDA-approved tools, enabling stable expression through random integration into the host genome. However, random insertion sites, variable copy numbers, and non-physiological expression driven by strong promoters can lead to insertional mutagenesis, fluctuating expression levels, and T-cell exhaustion (188). Subsequently, recombinant AAVs(rAAVs) gained attention for their low immunogenicity and high safety profile. Their single-stranded DNA genomes persist as episomes without integrating into the genome, making them suitable as homology repair templates for precise knock-in when combined with CRISPR–Cas9 (189). Nevertheless, rAAVs have a limited packaging capacity (~4.7 kb), and the episomal DNA is diluted in dividing cells (190). In recent years, non-viral delivery methods, primarily electroporation, have rapidly advanced. By co-delivering CRISPR ribonucleoprotein complexes with DNA repair templates, these methods enable site-specific integration, offer high editing efficiency and shorter production cycles, and avoid viral-associated risks (187). They are particularly suitable for precise insertion of smaller fragments (e.g., TCR or CAR sequences), but their insert size is limited (~1.5 kb), editing efficiency decreases with larger fragments, and large-scale manufacturing processes still require optimization.

Numerous preclinical studies have demonstrated the feasibility and potential of the CRISPR-Cas9 system for editing human T cells. For example, knocking out the CCR5 gene in CD4^+^ T cells confers resistance to HIV infection; knocking out CD7 in CD7 CAR-T cells prevents “fratricide” since T cells themselves express CD7 (191); and knocking out PD-1 in CD19 CAR-T cells enhances tumor clearance (192). As the technology matures, CRISPR-related clinical trials have also been initiated. For instance, CRISPR Therapeutics and Vertex launched a gene-editing trial for β-thalassemia(NCT03655678), and the University of Pennsylvania, in collaboration with Tmunity and the Parker Institute for Cancer Immunotherapy, initiated a CRISPR-edited T-cell cancer immunotherapy trial(NCT03399448). The accumulated gene-editing experience and clinical translation data in conventional T cells will provide crucial technical references and safety paradigms for the design and development of engineered Treg therapies. However, classical CRISPR-Cas nuclease technology relies on DNA double-strand breaks, a process associated with potential genomic instability risks such as chromosomal translocations, large deletions, and p53-mediated DNA damage response activation, limiting its broad clinical application (193). To overcome this fundamental constraint, a new generation of gene-editing tools has emerged. These technologies, born from a deep understanding and engineering of Cas protein functions, have developed along three complementary and increasingly sophisticated paths: precise DNA editing without double-strand breaks, dynamic RNA-level regulation, and sequence-agnostic epigenetic programming (183).

At the level of precise DNA editing, engineering of the Cas9 protein has yielded nickases (cutting only one strand) and catalytically dead Cas9 (dCas9). Building on these, base editing and prime editing technologies enable direct and precise modification of DNA bases without introducing double-strand breaks, greatly enhancing editing safety (193). Specifically, base editors fuse dCas9 or a nickase with a single-stranded DNA deaminase to efficiently achieve C•G to T•A or A•T to G•C conversions. Next-generation base editors (e.g., BE4max, ABE8e) exhibit extremely high editing efficiency and specificity in human cells and animal models (183). Prime editing represents a more versatile “search-and-replace” system capable of all 12 possible base conversions, as well as precise small insertions and deletions, completely avoiding double-strand breaks and associated risks, and lacking the bystander edits common in base editing, paving a new path for truly precise genome editing (194).

Beyond DNA, CRISPR–Cas-based RNA-targeting tools are also under development. Utilizing RNA-targeting Cas systems (e.g., RCas9, Cas13) allows for reversible knockdown, degradation, or real-time imaging of specific transcripts without altering the genome (195, 196). For example, RNA base editing achieved by fusing dCas13 to adenosine deaminase on RNA (ADAR) enzymes can reversibly correct disease-associated RNA mutations, providing new tools for dynamically regulating T-cell function (197). This post-transcriptional intervention avoids the permanent changes and potential risks of genomic editing, making it particularly suitable for therapeutic scenarios requiring transient or tunable functional modifications. Furthermore, dCas9-based epigenetic editing technologies have opened revolutionary avenues for programming T-cell fate and function. By fusing dCas9 with transcriptional activation/repression domains (e.g., CRISPRa/i) (198–202), histone modifiers (e.g., p300, TET1) (203, 204), or DNA methyltransferases (205), it is possible to programmably “write” or “erase” the chromatin state of specific genes without altering the DNA sequence, thereby precisely and reversibly upregulating or silencing key immunoregulatory genes. This epigenetic reprogramming capability makes it possible to finely “tune” the function of Tregs, potentially yielding more stable and controllable therapeutic cell products.

Above all, delivery platforms are evolving from viral systems toward non-viral systems, providing diverse and increasingly safe and efficient toolkits for next-generation Treg engineering. Concurrently, genome editing technologies such as CRISPR-Cas are transforming T cell therapy by emphasizing precision and safety. Together, these advances provide a robust arsenal to address current limitations and realize the potential of EngTregs. By precisely rewriting cellular blueprints, researchers can now design “smart” Tregs that exhibit enhanced stability, specificity, and durable function.

#### 6.4.4 Combination therapy: rational design and strategic integration

Long-term maintenance of Treg function post-adoptive transfer is critical for preventing the relapse of chronic diseases. Clinical studies of adoptive Treg therapy in T1D, however, have reported a sharp decrease in peripheral Treg counts post-infusion, potentially limiting therapeutic efficacy (156, 160). It is noteworthy that, while antigen-specific Tregs tended to exhibit prolonged persistence compared to polyclonal Tregs in murine models, their numbers ultimately diminished over time (179). Therefore, elucidating the mechanisms underlying this decline and determining whether long-term Treg survival is required, are pivotal for enhancing the overall therapeutic efficacy of Treg-based interventions. These challenges underscore the necessity of developing combination strategies that harness synergistic effects. Emerging evidence suggests that combined use of two or more immunotherapeutic agents can exert synergistic tolerance to improve therapeutic efficacy or reduce adverse reactions (206–209).

Current research is evolving from simple additive combinations of therapies toward intelligent engineering integration. Early combination approaches, such as co-administration of IL-2 and rapamycin (64), can expand Tregs but may incur β-cell toxicity (210), highlighting the risks of simplistic additive strategies. In the context of islet transplantation, the use of Tregs in conjunction with donor-recipient mixed hematopoietic chimerism may effectively promote transplant tolerance while circumventing the need for mTOR inhibitors (208). The new generation of strategies aims to achieve programmed integration of multiple functions within a single Treg product via genetic engineering. To avoid IL-2-mediated activation of inflammatory immune subsets, multiple gene-editing strategies have been developed to achieve Treg-specific targeting of IL-2 signals. One approach involves constructing synthetic orthogonal receptor-ligand pairs, in which engineering Tregs with orthogonal IL-2 receptors can only be activated by exogenously administered synthetic ligands (211). Another strategy integrates intracellular domains of the IL-2 signaling pathway (e.g., STAT3/STAT5-binding domains) into CARs. Studies demonstrated that incorporating STAT3/STAT5-binding domains within the cytoplasmic region of anti-CD19 CARs in conventional T cells significantly enhanced their *in vivo* persistence and anti-tumor efficacy (212). Collectively, these approaches may hold value for T1D therapy due to defective IL-2 signaling in Tregs within this disease context; however, their applicability in Treg therapies awaits experimental validation.

Recently, Wickham and Mueller engineered a Treg cell product termed GNTI-122, whose design logic employs a triple-precision editing strategy to address the multifaceted challenges of Treg therapy synergistically (209). First, using HDR-based gene editing to site-specifically integrate a strong promoter upstream of the endogenous FOXP3 locus, enabling physiological, stable, and high-level expression of this key transcription factor. This fundamentally solidifies the Treg lineage identity and suppressive function of the cells, preventing functional drift post-transfer. Second, introducing a TCR specific for IGRP endows the engineered Tregs with the precise ability to recognize autoantigens presented by local antigen-presenting cells within the pancreatic inflammatory milieu. This ensures their specific recruitment to the site of pathology and enables antigen-directed immunosuppression, overcoming the targeting deficiency of polyclonal Tregs. Finally, the innovative incorporation of a chemically inducible signaling complex (CISC). This system activates downstream IL-2/STAT5 signaling only upon administration of an exogenous small-molecule drug (e.g., rapamycin). This “on-demand” mechanism provides a dual advantage: it furnishes the infused Tregs with a potent, externally regulatable proliferative and survival signal, directly compensating for the intrinsic IL-2 response defect in T1D patient Tregs; and it grants clinicians precise control over the magnitude and timing of Treg expansion *in vivo* by managing the administration of the CISC activator, thereby substantially widening the therapeutic safety window and avoiding the non-specific activation of other immune cells associated with conventional IL-2 therapy.

The successful integration exemplified by GNTI-122 indicates that future combination therapy design should adhere to “modular” and “regulatable” principles. Beyond the aforementioned modules, additional functional modules that could be integrated to enhance EngTregs include:developing “off-the-shelf” cell therapies by knocking out MHC genes to reduce host rejection of allogeneic Tregs (213), overexpressing immunosuppressive cytokines (e.g., IL-10) (214), or genetically engineering Treg metabolic pathways (215). In conclusion, the future of combination therapy lies not in the simple co-administration of drugs but in the multi-gene, logic-based precision programming of the therapeutic cells themselves. Products like GNTI-122 mark the beginning of this paradigm. Subsequent research efforts should focus on optimizing individual functional modules, exploring safer and more controllable switch systems, and establishing rigorous preclinical and clinical evaluation standards matched to these intelligent cellular therapeutics to accelerate their translation into clinical application.

### 6.5 The clinical research landscape and translational challenges of Treg cell therapy in T1D

Prospective clinical studies serve as the core driving force behind the clinical translation of Treg therapy. As of July 2019, 51 clinical trials involving Treg cells were registered on ClinicalTrials.gov. These trials were summarized by Ferreira et al (216). With continued advancements in the field, the number of Treg cell clinical trials focused specifically on T1D had reached 14 by November 2025. Among these, six studies have been completed (NCT01210664, NCT02772679, NCT01827735, NCT03444064, NCT02691247, NCT02265809), four are actively recruiting participants (NCT06688331, NCT02932826, NCT06708780, NCT06427421), one has not yet started recruitment (NCT05973734), one has been withdrawn (NCT03236558), and two have an unknown status (NCT00173641, NCT03011021).

The completed trials primarily evaluated the adoptive transfer of polyclonal Tregs (e.g., NCT01210664, NCT03444064) and combination therapies with low-dose IL-2 (e.g., NCT02265809, NCT01827735). While the limitations of these approaches have been detailed previously, they collectively confirm the foundational safety of Treg therapy, thereby establishing a crucial groundwork for further investigation. Current research frontiers are focused on overcoming the key obstacles in translating Treg therapy from basic research to clinical application. First, patient heterogeneity and cell sourcing pose significant challenges to treatment accessibility. Patients with autoimmune diseases often receive long-term immunosuppressive treatments, which can compromise the number and function of their endogenous Treg cells. This makes it difficult to isolate and expand sufficient Tregs that meet therapeutic standards, representing a typical barrier in Treg transplantation trials (217). To address this, researchers are actively exploring alternative cell sources. For instance, umbilical cord blood (UCB)-derived Tregs have emerged as a promising option due to their more naive phenotype and greater proliferative potential (218). Notably, CD4 and CD25 markers are sufficient for their effective isolation from UCB (219). A clinical study (NCT02932826) evaluating the efficacy of ex vivo expanded UCB-Tregs for autoimmune diabetes is currently recruiting patients. Additionally, Tregs isolated from discarded thymic tissue obtained during pediatric cardiac surgery show considerable potential, with yields from a single donor comparable to those from adult peripheral blood (220). However, the clinical feasibility of this source requires further preclinical and clinical validation (220), and a study investigating thymus-derived Tregs in T1D has been withdrawn (NCT03236558), indicating remaining uncertainties for this pathway.

Second, refining therapy design toward greater precision is a key direction for improving efficacy. The field has placed strong emphasis on developing antigen-specific therapies. A clinical trial (NCT06708780) aiming to select antigen-specific Treg TCRs is currently underway. In the area of islet transplantation, another study (NCT06427421) is comparing the efficacy of recipient-derived Tregs versus donor bone marrow-derived immune cells in combination with islet transplantation to optimize immunomodulatory strategies. However, traditional pancreas or islet transplantation remains limited by donor scarcity and variable outcomes. In this context, new hope has emerged for numerous patients with long-standing, severe T1D in the form of Zimislecel (VX-880), Vertex Pharmaceuticals’ stem cell-derived, fully differentiated islet cell therapy, which offers the prospect of freedom from insulin dependence (221). This therapy also holds potential for future synergistic or complementary approaches with Treg therapy.

Of critical importance, the clinical translation of EngTreg therapies faces severe systemic challenges. Although engineered Treg therapies like ABA-201 are planned to initiate in 2025, as previously mentioned, no gene-engineered Treg therapy for T1D is currently registered on ClinicalTrials.gov, reflecting the significant gap between proof-of-concept and clinical implementation. In terms of safety, TCR-mediated off-target and cross-reactivity risks constitute a fundamental safety concern. Engineering TCRs is inherently complex, partly due to their native heterodimeric structure—introduced exogenous TCR subunits can mispair with endogenous TCRα or TCRβ chains, forming mixed TCRs with unknown specificity, thereby significantly increasing the risk of unpredictable off-target effects (216). Furthermore, even successfully designed high-affinity TCRs may cross-react with structurally similar self or foreign peptides, potentially leading to unintended autoimmune reactions or altered efficacy (222). While genome editing technologies offer potential solutions for precise TCR expression control, this path itself raises profound ethical considerations.

In terms of manufacturing scalability, the production process for EngTreg therapies is inherently complex, multi-step, lengthy, and costly. Achieving large-scale expansion compliant with GMP standards is a key constraint to broad accessibility. Notably, advanced manufacturing platforms with extensive experience in the CAR-T cell therapy field have widely adopted various systems to enhance process control and scale-up capability, ranging from semi-automated equipment (e.g., Miltenyi Biotec’s CliniMACS Prodigy, Cytiva’s Xuri system, ScaleReady’s G-REX) to fully automated integrated platforms (e.g., Lonza’s Cocoon Platform). These systems, utilizing closed operations and process standardization, help improve product consistency and reduce contamination risk, providing an important reference for future Treg scale-up production (223–225).

Regarding quality control and product release, although regulatory agencies have not stipulated a unified minimum cell viability threshold for cell therapy products, CAR-T cell clinical practice has established an important reference: clinical trials typically require a minimum cell viability of 70%, while commercial product release requires at least 80% viable cells (223). This standard provides a practical basis for Treg product quality specifications. Establishing comprehensive quality control and release standards throughout the entire process—from rigorous testing and qualification of starting materials (226) (e.g., patient cells) to precise control of critical process parameters during manufacturing—is fundamental to ensuring product batch-to-batch consistency, purity, and potency (223, 227). On the regulatory front, global regulatory agencies are actively working toward establishing harmonized frameworks for these advanced therapy products (227, 228). As living, genetically modified products, their long-term safety (e.g., tumorigenicity, insertional mutagenesis risk) and durability of efficacy are central to regulatory scrutiny. Some regulatory agencies (e.g., Japan’s PMDA) have introduced breakthrough review pathways like “Sakigake” to accelerate the approval of innovative therapies, but such pathways are also accompanied by stringent requirements for post-marketing studies to verify clinical benefits (229, 230).

In summary, although Treg therapy has achieved preliminary safety validation in the context of T1D, the comprehensive clinical translation of engineered Treg therapies still remains a considerable journey. Ensuring TCR specificity and safety, overcoming challenges in manufacturing, safety verification, and individualized application, and appropriately addressing associated ethical and societal issues are core problems that must be resolved to advance these next-generation treatments.

## 7 Discussion

This review underscores the pivotal yet compromised role of Tregs in the pathogenesis of T1D. Rather than a mere numerical deficit, Treg functional impairment within the inflammatory pancreatic islet microenvironment is identified as a central deficit to the breakdown of immune tolerance. This dysfunction stems from a complex interplay of genetic susceptibility (particularly SNPs affecting Treg-specific epigenetic landscapes and IL-2 signaling), epigenetic instability (e.g., Foxp3 TSDR methylation), metabolic dysregulation, and the disruptive influence of local inflammatory cytokines. Pancreatic Tregs exhibit unique adaptations, including a characteristic CXCR3^+^ effector signature revealed by transcriptomic and single-cell analyses, although their homing and suppressive capacity are often impaired in T1D. Advances in understanding Treg heterogeneity, stability, plasticity, and tissue-specific functions provide critical insights for therapeutic intervention.

Current Treg-based therapeutic strategies aim to correct the above dysfunction. Initial approaches, such as autoantigen vaccines and low-dose IL-2, aimed to induce or expand antigen-specific Tregs but faced limitations in efficacy and specificity. The field has since shifted toward more potent and precise interventions, namely adoptive Treg transfer and its advanced iteration, EngTregs. The foundational goals of EngTreg design—ensuring stable FOXP3 expression and conferring antigen specificity via TCR or CAR—are now being radically empowered by next-generation genome editing and delivery technologies. Although not discussed in detail in this review, it is undeniable that research on microbiome-mediated regulation of Treg function is rapidly advancing. Evidence indicates that gut microbiota directly modulate Treg cell differentiation, function, and stability through microbial metabolites, notably secondary bile acids (e.g., isoalloLCA) (231, 232)and short-chain fatty acids (e.g., butyrate) (232, 233). In T1D, gut dysbiosis—often accompanied by disrupted secondary bile acid metabolism and reduced levels of beneficial metabolites—may attenuate this positive immunoregulatory influence on Tregs (234–236). Consequently, interventional strategies aimed at restoring microbial balance or supplementing specific microbiota-derived metabolites, such as washed microbiota transplantation, selected probiotic administration, or direct metabolite supplementation, have emerged as promising therapeutic avenues for modulating Treg activity, reinstating immune tolerance, and ameliorating T1D progression (235, 237, 238).

The future of T1D immunotherapy lies in multifunctional, integrated EngTreg approaches that combine stable identity, precise antigen targeting, enhanced fitness, and microenvironmental adaptability. However, the clinical translation of such engineered Treg cell therapies still faces substantial challenges. Overcoming challenges related to TCR affinity/source selection, CAR target identification, ensuring long-term stability *in vivo*, and managing patient heterogeneity, will be essential. By leveraging a growing understanding of Treg dysfunction and harnessing modular genetic editing tools, the next generation of therapies holds the promise not merely of managing T1D, but of fundamentally resetting the immune system to achieve lasting remission or prevention.
